# Are Personal Resources and Perceived Stress Associated with Psychological Outcomes among Israeli Teachers during the Third COVID-19 Lockdown?

**DOI:** 10.3390/ijerph19095634

**Published:** 2022-05-05

**Authors:** Shiri Shinan-Altman, Inbar Levkovich

**Affiliations:** 1 Louis and Gabi Weisfeld School of Social Work, Bar Ilan University, Ramat-Gan 52900, Israel; 2 Oranim Academic College of Education, Kiryat Tiv’on 36006, Israel; inbar.lev2@gmail.com

**Keywords:** coping resources, COVID-19, depression, loneliness, perceived stress, resilience, teachers

## Abstract

Teachers’ psychological responses during a pandemic, such as COVID-19, play an important role in their adaptation to the new routine. This research aimed to explore the association between personal resources (sense of control, social support), perceived stress, and three psychological outcomes (resilience, depression, loneliness) among teachers during the third COVID-19 lockdown in Israel. A cross-sectional study was conducted among 208 teachers. Significant associations were found between perceived stress, resilience, depression, and loneliness. Sense of control was significantly associated with perceived social support. The research model was tested using Structural Equation Modeling. The model yielded appropriate indices of fit (χ2(10) = 10.31, χ2/df = 1.03, *p* = 0.413, NFI = 0.970, NNFI = 0.997, CFI = 0.999, RMSEA = 0.012, 95%CI RMSEA = 0.077), demonstrating that the model fits the data well. Findings suggest that in order to improve teachers’ psychological health during a virus outbreak, it is recommended to pay attention to their personal resources and perceived stress.

## 1. Introduction

During the COVID-19 pandemic, several studies have reported elevated levels of stress, anxiety, and depression among individuals [1,2,3]. Specifically in the third lockdown, which lasted 42 days, there were strict lockdown regulations in Israel. These included remaining within 1000 m of one’s home, prohibitions against visiting others in their homes and the banning of gatherings of more than 20 people. By this time, the number of fatalities from COVID-19 in Israel had reached 3346, and the third wave had peaked with 1444 deaths in the former month [1]. Studies conducted during the third lockdown in Israel have found depression, anxiety, and psychological distress among the general population as a result of COVID-19 [1,4].

In Israel, the education system was closed down during the three lockdowns that were imposed in the country, following the outbreak of the COVID-19 pandemic. Each of these lockdowns lasted for about eight weeks. During the three lockdowns, and also in periods between them, teachers had to shift from frontal teaching to online distance teaching. While schools were closed during the lockdown periods, questions arose regarding how teachers could best provide students with help and support during these challenging times and how to maintain successful teacher -student relationships. Many teachers indicated that the transition to the use of remote learning technology and digital platforms—often with a lack of technical support and without prior training—added to their perceived stress [5]. Teachers reported that preparing for the lessons became complicated and challenging, as they had to create or find new suitable materials for distance teaching, whilst overcoming technical problems [6,7]. An additional source of teachers’ perceived stress stemmed from the need to create a balance between home space and professional space [8]. Many teachers had to share their home-based workspace with other family members studying or working from home. The need to simultaneously support their students as well as their own children was another source of stress among teachers [5,8]. This prolonged exposure to a stressful situation may create negative psychological outcomes among teachers [9].

In this study, we concentrated on three psychological outcomes among teachers during COVID-19: resilience, depression, and loneliness. Resilience is one’s ability to cope with difficulties and to be flexible enough in adapting to new demands in stressful situations [10]. It was suggested that resilience predicts better coping with a variety of stressful situations, such as COVID-19 [11]. A study conducted in Israel among 300 participants examined the effect of resilience and well-being on symptoms of distress during the first lockdown of COVID-19. This study found that higher resilience and well-being contributed to reduced distress symptoms and a decreased sense of danger [12]. Depression is one of the most common psychiatric disorders in the Western world. The prevalence of depression in the general population ranges from 3% to 10% [13]. Among teachers, studies conducted during COVID-19 indicated higher levels of depression, anxiety, and distress [6,14]. A study conducted in Spain among 1600 teachers found symptoms of depression in 32.2% teachers [15]. Loneliness is defined as a subjective feeling of dissatisfaction following a lack of satisfying interpersonal relationships [16]. The COVID-19 crisis has forced many countries to implement social distancing policies and lockdowns at different times [2,17]. Studies examining the impact of loneliness on people’s lives during quarantine periods have reported that loneliness was associated with suicidal thoughts among adults in the United States [18,19]. In addition, a study conducted in April 2020 during the COVID-19 period, which examined 634 language teachers from Europe and North America, found positive associations linking loneliness with sadness, and anger [5]. It was further found that young people, women, single people, divorced people, and people with emotional regulation difficulties reported a higher level of loneliness [20,21].

This study was based on the stress and coping theory [22]. According to this theory, coping involves persistently changing cognitive and behavioral efforts aimed to manage internal and/or external demands that are assumed as exceeding or taxing one’s resources. This model proposes that the psychological and physical health outcomes of coping with chronic and intense stressors are influenced by personal resources, stressor appraisals, and psychological and physical outcomes. Following this theory [22], we assumed that adjustment to the abovementioned psychological outcomes (resilience, depression, loneliness) depends mainly on coping resources and perceived stress. According to the revised version of the job demands–resources model it is important to consider internal resources (called ‘personal resources’) in addition to external resources used to meet the job demands [23]. Accordingly, in this study, two coping resources were examined among teachers: sense of control (internal resource) and perceived social support (external resource) because these two coping resources have been found in the literature to be associated with psychological outcomes among teachers [8,24]. Sense of control refers to the real or perceived control of individuals over their life and their perceptions regarding their ability to cope with stressors [25]. Perceived social support is defined as the perception that one is part of a social network of people (e.g., friends, family) who care for one’s safety [26].

Apart from the direct influence of coping resources and perceived stress on adjustment to stressful events, several complex models have been suggested. One of these models is the mediating model [27], known also as the deterrence model. According to this model, internal and external coping resources decrease perceived stress, which in turn decreases maladaptive psychological responses to stressful situations.

To sum up, the COVID-19 crisis, together with the intermittent closure of the education system and the shift to online distance teaching, have raised the levels of emotional distress among some teachers [6]. To date, the Israeli education system has undergone three such closures. To our knowledge, no study has yet examined how Israeli teachers coped during the third lockdown, after nine months of coping with the COVID-19 crisis in Israel. Therefore, the aim of this study was to examine a comprehensive model that integrates the interrelationships among perceived stress, personal resources (sense of control, perceived social support), and three psychological outcomes (resilience, depression, and loneliness) among teachers during the third COVID-19 lockdown in Israel. In addition, we examined indirect relationships, involving perceived stress as an independent variable, personal resources as mediators, and resilience, depression, and loneliness as the outcome variables.

Specifically, the following hypotheses were tested:

**H1.**  *Higher levels of perceived stress, and lower levels of personal resources (sense of control, perceived social support) will be directly associated with lower resilience, higher depression, and higher loneliness.*

**H2.**  *Personal resources (sense of control, perceived social support) would mediate the association between perceived stress and three psychological outcomes (resilience, depression, and loneliness).*

## 2. Materials and Methods

### 2.1. Participants

A total of 208 teachers working in public schools throughout Israel participated in the study; 180 were women (86.5%) and 28 were men (13.5%). Sample size was calculated using the G*Power software (version 3.1.9.7) [28,29]. For a regression analysis with 7 predictors, an effect size of f^2^ = 0.145 (equals the minimum R^2^ of 0.17 that was found), α = 0.05, and power = 0.95, the required sample size is 158 participants. All higher effect sizes (i.e., higher R^2^), require smaller samples. The criterion for inclusion was being a teacher working in the Israeli school system. The criterion for exclusion was not filling the whole questionnaire. The participants ranged in age from 24 to 65 (Mean = 43.4, SD = 10.1). Their mean teaching experience was 14.8 years (SD = 10.5). Most of the participants were married (n = 174, 83.7%) and had an average of 2.5 children (SD = 1.1). Most of the teachers (59.1%) had a Master’s degree; 135 participants worked in middle and high schools (64.9%).

### 2.2. Procedure

The present study was approved by the university’s Ethics Committee (Approval no. 99-21). A convenience sampling method was used to recruit the study participants. The research participants were invited to take part in a study focused on teachers’ coping during the third COVID-19 lockdown. The teachers were recruited mainly through internet forums and social media outlets (Facebook pages for teachers). A total of 208 teachers visited the online survey and filled out the whole questionnaire. Another nine were excluded due to incomplete questionnaires. The study was conducted between January 2021 and February 2021. This period of time reflects the third lockdown in Israel. During this time, all schools were closed and teachers were asked to teach via online platforms. The survey’s introductory page stated explicitly that proceeding to the questionnaire would signify consent to participate.

### 2.3. Measures

#### 2.3.1. Dependent Variables

*Resilience* was measured using the Brief Resilience Scale (BRS) [30], which assesses one’s ability to ‘bounce back’ after experiences of distress and includes positive and negative items. Participants were asked to indicate the extent to which they agreed or disagreed with each item on a 5-point Likert scale ranging from 1 = strongly disagree to 5 = strongly agree (e.g., “It does not take me long to recover from a stressful event”). After reversing the negative statements, a mean score was calculated; a high score indicated greater levels of resilience (Cronbach’s α = 0.83).

*Depression* was assessed using the Symptoms of Depression Questionnaire (Center for Epidemiological Studies Depression, CESD–10) [31], which is composed of 10 items and is a shortened version of the original 20-item instrument [32]. Participants were asked to rate the intensity of their experiences during the previous week on a 4-point Likert-type scale ranging from 0 = never to 4 = to a great extent (e.g., “I felt depressed”). After reversing the positive statements, a mean score was calculated; a high score indicated higher levels of depression (Cronbach’s α = 0.85).

*Loneliness* was assessed using the Revised U.C.L.A. loneliness scale (R-UCLA) [33], a shortened version of the original questionnaire [34], which was validated in Hebrew [35]. The questionnaire includes three items relating to the severity of general loneliness. Participants were asked to rate each statement on a 5-point Likert-type scale, ranging from 0 = not at all to 4 = very much (e.g., “To what extent do you feel isolated from others?”). A mean score was calculated; a high score indicated higher levels of loneliness (Cronbach’s α = 0.91).

#### 2.3.2. Independent Variables

*Sense of control* was assessed using a 7-item scale measuring the ability to have control over issues in one’s lives [25]. Participants were asked to rate the extent to which they agreed or disagreed with each statement on a 7-point Likert scale ranging from 1 = strongly disagree to 7 = strongly agree (e.g., “I can do almost anything I decide”). A mean score was calculated; a high score indicated greater levels of sense of control (Cronbach’s α = 0.73).

*Perceived social support* was assessed using the Multidimensional Scale of Perceived Social Support [36], a 12-item scale validated in Hebrew. Participants were asked to rate the extent to which they agreed or disagreed with each statement on a 7-point Likert scale ranging from 1 = strongly disagree to 7 = strongly agree (e.g., “My friends really try to help me”). A mean score was calculated; a high score indicated greater levels of perceived social support (Cronbach’s α = 0.92).

*Perceived stress* was measured using a single item that seeks to assess participants’ subjective stress experience: “Please rate your stress level during the past week”. This item has been widely used in past studies [37]. Participants were asked to indicate the extent of their stress on a 10-point Likert scale ranging from 1 = no stress to 10 = very high stress; a high score indicated higher levels of perceived stress.

*Personal and professional background* included gender, age, years of education, marital status, number of children, and teaching seniority.

### 2.4. Statistical Analyses

Data were analyzed using SPSS and AMOS (ver. 27). Descriptive statistics were used to describe the participants’ demographic characteristics and the research variables. Pearson correlations were calculated to assess the associations between the research variables. No missing data were noted. The variable of social support was negatively skewed (skewness= −1.64, SE = 0.17) and was thus exponentially transformed. The variable of loneliness was positively skewed (skewness = 0.53, SE = 0.17) and was thus logarithmically transformed. No meaningful outliers were noted. Independent continuous variables were standardized. The research model was tested with path analysis, with maximum likelihood estimation (ML), using AMOS 27. Model fit was assessed with five indices. Chi-square and the normed chi-square (χ2/df) tests were used to assess the model’s overall fit and parsimony. Normed chi-square values of ≤2.0 indicates a good fit. We used also comparative fit index (CFI), normed fit index (NFI), and non-normed fit index (NNFI), which are incremental fit indices. We employed the root mean-square error of approximation (RMSEA and its confidence interval), which measures the discrepancy per degree of freedom and indicates the model’s absolute fit. CFI, NFI, and NNFI scores of >0.95 and RMSEA values of <0.06 indicate a good model fit [38]. In addition, indirect effects were evaluated, within the path analysis model, by using a bootstrapping test (5000 bootstrap samples), and 95% bias-corrected confidence intervals (CI), in order to evaluate the statistical significance of the indirect paths.

## 3. Results

### 3.1. Descriptive Statistics and Correlations between Study Variables

As can be seen in Table 1, the mean score for perceived social support was relatively high and the mean scores for sense of control and resilience were relatively moderate to high. The mean score for perceived stress was relatively moderate, and the mean scores for depression and loneliness were relatively moderate to low.

According to Table 1, loneliness was significantly and positively associated with depression—namely, the more the participants experienced a sense of loneliness, the more depression they reported feeling. In addition, the higher the participants’ sense of control was, the greater their perceived social support, and the lower their perceived stress—the lower their feelings of depression and loneliness were. Significant negative moderate associations were found linking depression and loneliness with resilience—namely, the lower the participants’ feelings of depression and loneliness were, the higher their resilience was. In addition, participants with higher levels of sense of control and lower levels of perceived stress, reported higher levels of resilience. Finally, higher levels of sense of control were related with higher levels of perceived social support, and with lower levels of perceived stress. No significant associations were found linking perceived social support with either resilience or perceived stress.

Prior to assessing the research model, the associations between the background characteristics of the teachers and the research variables were examined, to identify control variables for the model. Significant correlations were found between the teachers’ ages and: perceived stress (r = −0.29, *p* < 0.001), depression (r = −0.23, *p* < 0.001), and loneliness (r = −0.19, *p* = 0.006), so that younger teachers reported higher levels of perceived stress, depression, and loneliness. Thus, the research model was assessed while controlling for the teachers’ ages. Controlling for the teachers’ ages controls for their seniority in teaching as well, as the correlation between these two variables was high (r = 0.79, *p* < 0.001).

In addition, depression scores were found higher for teachers working in middle and high schools (M = 1.16, SD = 0.53) than for teachers working in elementary schools (M = 0.98, SD = 0.57) (t(206) = 2.29, *p* = 0.023). Other differences were not found significant, and thus teachers’ ages and students’ age levels (0—elementary school; 1—middle and high school) were controlled for.

### 3.2. The Study Model

**Figure 1:**
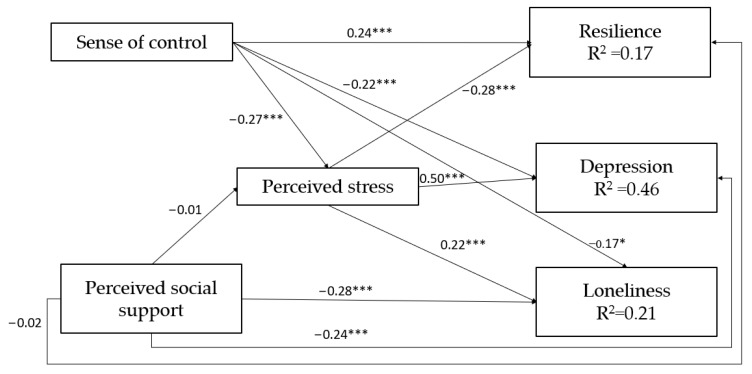
The study model. Note: All independent and mediating parameters are standardized. * *p* < 0.01, *** *p* < 0.001.

The research model presented in Figure 1 yielded appropriate indices of fit (χ2(10) = 10.31, χ2/df = 1.03, *p* = 0.413, NFI = 0.970, NNFI = 0.997, CFI = 0.999, RMSEA = 0.012, 95%CI RMSEA = 0.0, 0.077), demonstrating that the model fits the data well. As can be seen in Figure 1, the regression model explained 17% of the variance in resilience, 46% of the variance in depression, and 21% of the variance in loneliness.

A significant association was found between sense of control and perceived stress, such that a higher sense of control was related with lower perceived stress. No relationship was found between perceived social support and perceived stress. Further, significant associations were found between sense of control and the three dependent variables—namely, the higher the participants’ sense of control was, the higher their resilience was, and the lower their depression and loneliness levels were. Similarly, significant negative direct associations were found linking perceived social support with depression and loneliness. No significant association was found between perceived social support and resilience. Finally, significant associations were found between perceived stress and the three dependent variables—namely, participants with higher levels of perceived stress reported lower resilience levels and higher levels of depression and loneliness.

As can be seen in Figure 1 and Table 2, perceived stress served as a mediating variable between sense of control and the three dependent variables. That is, higher sense of control was related with lower perceived stress, which in turn was related with higher resilience, lower depression, and lower levels of loneliness.

No significant association was found between perceived social support and perceived stress. Therefore, no mediation was examined for perceived social support and the outcome variables.

## 4. Discussion

The aim of this study was to explore the associations among personal resources (sense of control, perceived social support), perceived stress, and three psychological outcomes (resilience, depression, and loneliness) among teachers during the third COVID-19 lockdown in Israel. According to our findings, a negative association was found between both coping resources and teachers’ depression and loneliness—namely, participants with higher levels of coping resources reported lower levels of depression and loneliness. These findings are supported by previous studies suggesting that coping resources may be protective factors in times of stress and may help promote individuals’ psychological health [39]. In a study conducted among teachers in Jordan during the COVID-19 crisis, about one-third of the participants reported experiencing anxiety, depression, and stress, noting that social and family support helped them and were a vital resource in their coping [40]. Similarly, a study conducted among teachers in China found that sense of control and perceived social support were associated with participants’ emotional state [41]. It should be noted that the COVID-19 crisis belongs to the phenomenon of “Shared Traumatic Reality” (STR). This state refers to the shared reality of professionals in the field of education and care: that they, together with their students or patients, live and/or work in shared areas that endanger their personal safety [42]. The COVID-19 pandemic is a new STR for teachers who experience the fear and danger of the virus and are concerned for their family members, and at the same time must provide support and help for their students. In this situation, these teachers need a great deal of coping resources—both for themselves and in order to provide others with support.

The findings of the current study indicate that the more teachers reported a sense of control, the higher their reported resilience levels. Sense of control is an internal resource which allows to cope with stressful events more effectively and to experience fewer negative consequences (e.g., depression, anxiety) caused by the exposure to the stressor [22]. A sense of control helps maintain a sense of resilience when coping with stressful situations and is positively associated with positive mental well-being [43]. A qualitative, longitudinal study conducted among U.S. teachers showed that teachers felt a better sense of control over time during COVID-19 [44]. Researchers suggest that resilience develops and strengthens over time dynamically, as a result of the individual’s positive coping strategies [45,46,47].

Our findings indicted that perceived stress served as a mediating variable in the relationship linking sense of control with resilience, depression, and loneliness. Perceived stress is the result of a person’s appraisal of a stressor as threatening or non-threatening. The person evaluates what can be done, if at all, to overcome the stressful situation and improve his/her mental well-being [22]. Assessing the stressful situation largely defines the coping strategies a person chooses as well as the coping outcomes [22]. The mediating role of perceived stress found in this study is in line with a study conducted among 1100 Israelis during the second lockdown period, which found that perceived stress mediated the association between participants’ perceived state of health and their emotional state [48]. It might be that people with a higher sense of control may experience lower perceived stress, perceive the situation as less challenging, and experience a lower sense of threat. These perceptions may create lower levels of depression and loneliness and higher levels of resilience. This finding is consistent with the stress and coping model [22], indicating the central role of both resources and perceived stress.

In this study, age was found to have a significant negative association with perceived stress, depression, and loneliness. Thus, younger teachers reported higher levels of these variables compared to older teachers. This finding is consistent with studies conducted in Israel and in other countries. In a study that included 46,000 participants aged 16–99 from 237 countries, it was found that loneliness levels were higher for younger individuals compared to older individuals during COVID-19 [49]. A study conducted among teachers in Spain indicated that the highest levels of depression and stress were found among teachers aged 23–35 [15]. Indeed, younger teachers may have less experience with difficult life events, and at the beginning of their professional career their work is more intensive and demanding, which may affect them emotionally [15,49]. In addition, during the quarantine period younger teachers were required to provide care for their young children and/or to cope with spouses whose employment status sometimes changed abruptly (working from home, furloughed, or unemployed). These factors may also have affected younger teachers’ psychological status.

Several potential shortcomings should be acknowledged. First, we used online questionnaires because of the limitations of social distancing restrictions; therefore, the response rate and reasons for refusal are unknown. Second, we applied a cross-sectional design and, therefore, bidirectionality of the associations among the variables cannot be ruled out and caution is advised when inferring causality. Caution is also advised in this regard when interpreting the results of the mediation model. Longitudinal studies with larger groups of teachers are therefore needed in order to develop a deeper understanding of the nature of the relationships examined in this study. Third, we have no information whether the associations between study variables found during the pandemic would be different (or probably the same) during the normal time before the pandemic. Additionally, probably the same associations could be found in a general population or other occupational groups. Fourth, only one instrument was validated in Hebrew. Fifth, other aggravating factors that may be responsible for the negative effects, such as a history of mental disorders, were not investigated in this study. Finally, because the current pandemic is dynamic and its impact is lasting, the emotional state of teachers should be examined across time.

## 5. Conclusions

This study is the first to identify factors contributing to psychological outcomes among teachers during a third lockdown of COVID-19, after nine months of coping with COVID-19 in Israel. Practically, the study findings emphasize that teachers need control during a pandemic. In addition, due to the research findings indicating higher levels of emotional distress among younger teachers, it is suggested to provide more support for younger teachers and appoint senior teachers who can educate them and help them cope in times of crisis. Intervention programs should include means for increasing sense of control and social support among teachers, in order to improve their coping, and ultimately decrease negative psychological outcomes during a pandemic outbreak. In order to develop a sense of understanding and advocacy, stakeholders, including parents, school district officials, and community partners need to know the mechanism that may lead to decreases in teachers’ well-being. In order to improve teachers’ psychological health during a virus outbreak, it is recommended to pay attention to their personal resources and perceived stress.

## Figures and Tables

**Table 1:** Correlates, means, SDs, and ranges of study variables (*n* = 208).

Variables	Mean (SD)	2	3	4	5	6
1. Resilience	3.54 (0.74)	−0.42 ***	−0.25 ***	0.32 ***	0.07	−0.35 ***
2. Depression	1.10 (0.55)		0.37 ***	−0.44 ***	−0.34 ***	0.39 ***
3. Loneliness	1.42 (1.13)			−0.31 ***	−0.33 ***	0.29 ***
4. Sense of control	5.04 (0.90)				0.28 ***	−0.29 ***
5. Perceived social support	4.45 (0.60)					−0.07
6. Perceived stress	5.51 (2.57)					

*** *p* < 0.001.

**Table 2:** Indirect effects between sense of control and the dependent variables (*n* = 208).

Dependent Variable	Indirect Effect	SE	95%CI	*p*
Resilience	0.08	0.03	0.03, 0.14	<0.001
Depression	−0.13	0.04	−0.20, −0.07	<0.001
Loneliness	−0.06	0.02	−0.12, −0.02	<0.001

## Data Availability

The data that support the findings of this study are available from the authors upon reasonable request.
